# Quality of life and functional outcomes in tongue cancer patients: a long-term, prospective, comparative study

**DOI:** 10.4317/medoral.26228

**Published:** 2023-10-12

**Authors:** Sebastian Rios-Gonzalez, Susana Heredero-Jung, Juan Jose Ruiz-Masera, Angel Martínez-Sahuquillo-Marquez

**Affiliations:** 1 Department of Oral and Maxillofacial Surgery. Reina Sofia University Hospital, Cordoba, Spain; 2 Department of Stomatology. University of Seville, Seville. Spain

## Abstract

**Background:**

The tongue has an indispensable role in communication, swallowing and breathing. Tongue cancer treatment involves direct resection of the tumor and surrounding tissue, which can limit many essential functions of the tongue. There are few patient-reported quality of life studies involving tongue cancer exclusively. There is also a lack of data on the outcomes of quality of life regarding different reconstructive methods, adjuvant non-surgical therapies and other predicting factors. Our objective is to assess the quality of life, functional status, and predicting factors in patients with tongue cancer up to one year after surgical resection.

**Material and Methods:**

Thirty-six patients with tongue cancer were prospectively identified between October of 2017 and January 2021. Patients were examined before and one, three, six and twelve months after surgical resection with the validated University of Washington Quality of Life questionnaire (UW-QOL). Data collection included patient age, sex, TNM staging, size of resection, neck dissection, tracheostomy, reconstructive method and adjuvant therapies. Outcome scores were compared using the Friedman test. Multiple linear regression analysis was used to identify the predictors of quality of life and functional status.

**Results:**

The use of UWQOL scores as dependent variables revealed the following predicting factors: age, tobacco use, radiotherapy, chemotherapy, reconstruction method and neck dissection.

**Conclusions:**

The most relevant findings in our study are that flap reconstruction becomes increasingly necessary when a glossectomy resection is over 45 mm, in order to maintain tongue function. We established that the reconstructive flap type does not influence quality of life in the long term. Also, we have found that cervical sentinel node biopsy provides better quality of life over neck dissection in the first 3 months after surgery.

** Key words:**Quality of life, tongue cancer, microsurgical reconstruction, treatment outcome.

## Introduction

Tongue cancer is the most common type of tumor of the oral cavity (1). The most frequent type is squamous cell carcinoma (1,2). The prevalence of tongue cancer varies from 3% to 90% depending on the country, with a clear rise in incidence in the last few years (2). Toxic habits such as smoking and alcohol consumption contribute to the pathogenesis, as well as Human Papilloma virus infection (HPV) (1). Tongue cancer is considered the deadliest type of oral cancer, with a mortality rate of over 40% within the first 5 years of diagnosis (2).

The top priority of surgeons is to save the patient’s life, therefore radical ablations are required in order to completely remove the cancer with safe margins (3). This results in significant deformities and morbidity (3). The second priority of surgeons is to reconstruct the part of the body that was resected, in our case the tongue. This reconstructive phase also follows certain priorities: to protect vital structures, to restore function and to provide an aesthetically pleasing result (3).

The tongue serves a host of functions that are vital to our everyday activities such as eating, speaking and breathing (2). Ablative resections create an impairment to the functions of the tongue that we must strive to counter with reconstructive surgery (4). The failure to restore the function of the tongue can condemn a patient to traqueal breathing, enteral feeding and incomprehensible speech (4).

The reconstructive techniques used after glossectomy depend on the size of the resection (2,4). The question for many surgeons becomes whether to reconstruct the tongue with direct closure for small resections or reconstructive flaps in case of larger resections, but the boundary to decide which is best is still to be determined, and it depends in part to the surgeon’s experience and reconstructive philosophy (4). The flaps used for reconstruction of the tongue are classified into different types according to their site of harvesting. The main two types that are used in our department are: Pedicled flaps (Submentalis Flap, Supraclavicular flap, and Pectoralis major flap) and free flaps (Anterolateral thigh [ALT] flap, Profunda Artery femoris Perforator [PAP] flap, Superficial Circumflex Iliac Perforator [SCIP] flap and the radial forearm flap) (5).

There is a lack of essential data of functional and quality of life outcomes of tongue cancer patients who underwent surgical resection and adjuvant therapy (6). The purpose of our study is to provide objective, long-term, patient-reported data on the function and quality of life before and after treatment of tongue cancer patients.

## Material and Methods

- Study design

This was a prospective, comparative and consecutive cohort study.

- Study setting

The present study was conducted at the Oral and Maxillofacial department of the Reina Sofia University Hospital in Cordoba, Spain. The study complied with the declaration of Helsinki and was approved by the ethics committee of the Andalusian Health Service (approval number: 3736 - May 2018). All patients were informed of the purpose of the study and signed an informed consent form. Data was collected using the Spanish version of the University of Washington Quality of Life (UW-QOL) questionnaire, version 4 (7). This questionary is used to evaluate the wellbeing and practical capabilities of tongue cancer patients during their surgical treatment. The initial assessments were conducted in the pre-surgical appointment with the lead surgeon. The follow-up assessments were done at one, three, six and twelve months after the surgery. Data was collected from October 2017 to February 2021, upon receipt of eligible candidates.

- Study participants

The inclusion criteria were are follows: 1. being newly diagnosed as having primary tongue cancer, 2. seeking to undergo surgical resection, 3. being above 18 years old, 4. being diagnosed at the Reina Sofia University Hospital, 5. histopathological diagnosis of squamous cell carcinoma (SCC). The exclusion criteria were as follows: 1. having a history of head and neck cancer, 2. having undergone previous resections of the tongue or oropharynx, 3. having a history of tongue dysfunction before diagnosis of tongue cancer, 4. having a history of dysphagia or dysphonia, 5. having undergone chemotherapy or radiotherapy before current diagnosis, 6. having a recurrence or relapse of the cancer in the one-year follow up period, 7. patient rescinding the informed consent agreement, 8. being lost to follow-up consultations. The eligible candidates were scheduled for follow-up visits with their lead surgeons after one, three, six and twelve months after surgery, respectively. Every eligible participant served as their own matched control before and after surgery.

- Reconstruction

Following a biopsy-proven cancer diagnosis, the stage of the disease was determined using the American Joint Committee on Cancer - TNM (AJCC-TNM) classification (8), and presented to the head and neck cancer multi-specialty committee of the Reina Sofia University Hospital to determine appropriate treatment. The patient’s demographics, history of tobacco and alcohol consumption, comorbidities, and adjuvant therapy were recorded. All patients in our study had surgical resection which involved various degrees of glossectomy. Reconstruction of the tongue was done according to the criteria of the lead surgeon, with small defects reconstructed by direct closure and larger defects requiring pedicled or free flaps to reconstruct. Management of neck lymph nodes was done according to T-status (tumor size): cervical sentinel-node biopsy was done in T1 and T2 patients if there was no evidence of neck dissemination found in the clinical and radiological analysis (N0) (2), no selective unilateral dissection was performed. Neck dissection was performed in all patients with T3 or T4 tumors or if there was evidence of neck dissemination in the clinical or radiological examination.

- Variables and data collection

The study had one main outcome variable: the patients’ overall quality of life according to the UW-QOL score. This score can be divided in two sub-categories: physical function which is based on the mean scores of chewing, swallowing, speech, taste, saliva and appearance, and its socio-emotional function which is based on anxiety, mood, pain, activity, recreation and shoulder function. In addition, the patient’s demographics, medical history, and toxic habits were recorded. After obtaining informed consent, every patient was given the questionnaires in the five occasions detailed previously.

- Statistical analyses

To compare the patients’ quality of life before and at one, three, six and twelve months after surgical treatment, centered on the average scores documented from their UWQOL questionnaires, the Friedman test was used if the scores showed non-normal distribution. Wilcoxon signed-rank test was performed to detect significance in differences between each pair in the comparison groups. The overall recorded scores from the UWQOL questionnaire were used as continuous dependent variables against preset categorical independent predictors (i.e., age, gender, drinking habit, smoking habit, tumor site, tumor size, and reconstructive technique). The stepwise regression method of multiple linear regression analysis was used to identify the predictors of quality of life and functional status at five different time points, namely before the surgery, one month, three months, six months, and twelve months after surgery from each of three questionnaires. The Physical function sub-category scores were used to identify the predictors of functional status. Moreover, the socio-emotional sub-category scores were used as dependent variables to identify the predictors of quality of life. SPSS software was utilized to for statistics analysis (IBM Corp. Released 2012. IBM SPSS Statistics for Windows, Version 25.0, Armonk, NY: IBM Corp.) at a 95% significance level.

## Results

In total, 44 tongue cancer patients were examined in this study. Of these, 8 patients were excluded (6 due to exitus within the first year of follow-up, 2 due to recurrence of tongue cancer). Eventually, 36 (81.0%) tongue cancer patients fulfilled the eligibility criteria for the present study. Table 1 summarizes the patient’s demographics, medical history, and lifestyle parameters. Of the 36 patients included in this study 16 (44.0%) were male and 20 (55.6%) were female. The mean age of the participants was 61.0 ± 4.2 years, and the age range was between 32 and 89 years. Regarding lifestyle parameters, 16 (44.2%) of the participants reported that they smoked tobacco and 11 (31.1%) reported that they had daily alcohol consumption.

Regarding medical history, 15 participants (41.6%) were diagnosed with stage I tongue cancer, 8 (22.2%) were diagnosed with stage II, 3 (8.3%) with stage III and 10 (27.7%) with stage IV. Postoperative radiotherapy was required in 20 (55.6%) study participants, only patients with stage I tongue cancer with free margins in the histological examination of the surgical resection and no evidence of cervical dissemination were excluded. However, only 3 (8.0%) patients required postoperative chemotherapy, all of them with stage IV disease. None of the patients required adjuvant radiotherapy or chemotherapy.

Tumor size ranged from 2 to 45 mm, with 18 (50.0%) being less than 20 mm, 15 (41.7%) being between 20 and 40 mm, and 3 (8.3%) being more than 40 mm. The size of surgical resection (maximum length of the sample) ranged from 19 to 110 mm, with only 1 (2.1%) being less than 20 mm, 22 (61.1%) being between 20 to 50 mm and 13 (37.8%) being over 50 mm. The types of resections were partial anterior glossectomy in 20 patients (55.6%), partial posterior glossectomy without tongue tip resection in 5 patients (13.8%), hemiglossectomy in 5 patients (13.8%) and total glossectomy in 6 patients (16.6%). Tracheostomy was required in 21 patients (58.3%). Neck dissection was performed according to TNM staging (8); with sentinel node biopsy performed in 11 patients (30.6%), unilateral modified radical neck dissection in 17 patients (47.2%), unilateral radical neck dissection in 1 patient (2.8%), and bilateral neck dissection in 7 patients (19.4%). Postoperative infection of the head and neck site occurred in 6 patients (16.7%). Reintervention within the postoperative hospitalization period was required in 8 patients (22.2%). The length of hospital stay ranged between 2 to 43 days, with 13 patients (36.1%) staying less than 7 days, 17 patients (47.2%) staying between 7 to 21 days, and 6 patients (16.6%) staying over 21 days.

Concerning reconstructive techniques, 20 patients (55.6%) were treated with direct closure after partial glossectomy, mainly in patients with T1 and T2 tongue cancer (<45 mm of resection). A free flap was needed for reconstruction in 14 patients (39.0%), most of them with large resections of the tongue (>45 mm). The most common type of free flap used was the PAP flap (5), used in 9 patients (25.0%), then the ALT flap used in 4 patients (11.0%), then the SCIP flap used in 1 patient (3.0%). A pedicled flap was used for reconstruction in 2 patients (6.0%); the Pectoralis major flap was used in 1 patient (3.0%) and a Submentalis flap was also used in 1 patient (3.0%).

**Table 1:**
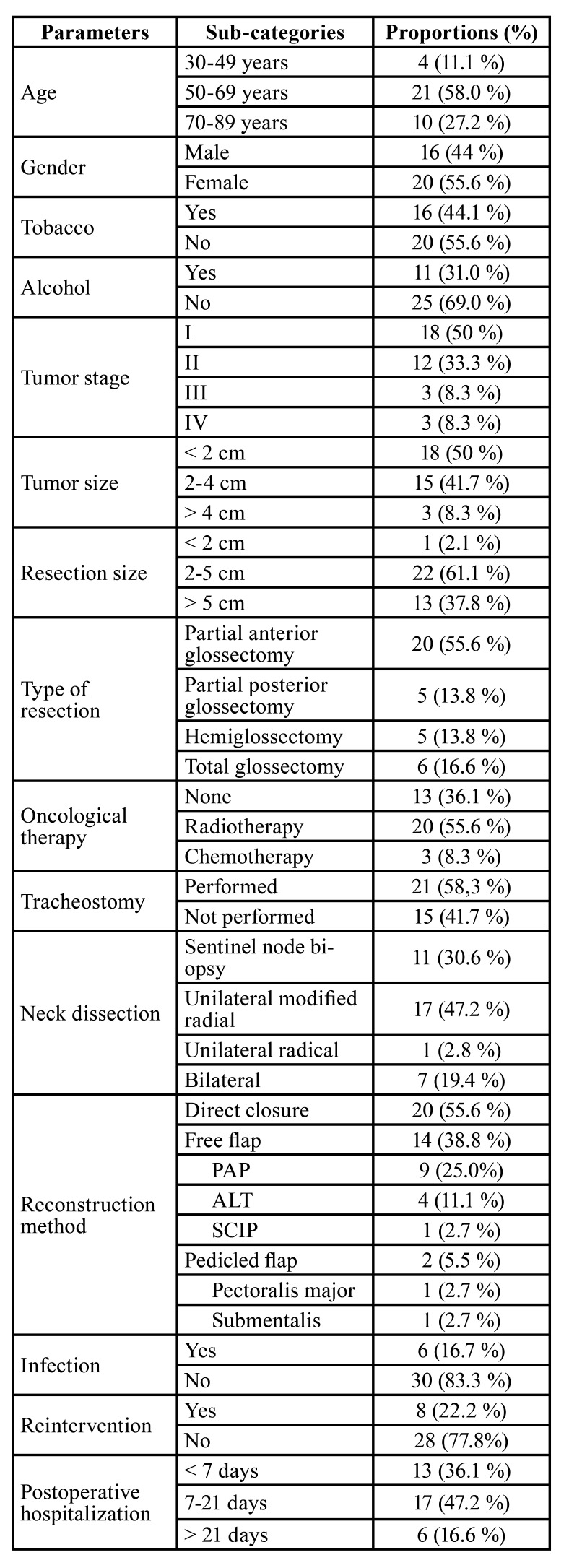
Patient demographics, medical history and lifestyle parameters.

Table 2 summarizes the comparison of the patients’ quality of life and functional status before and one, three, six and twelve months after surgical treatment. There was a significant difference in the physical subset scores before and one, three, six and twelve months after surgery (F= 69.8, *p*<0.01). The mean physical score at twelve months [77.8] was lower than the score before surgery [91.8], but it was higher that the scores at one [61.9], three [61.8] and six [68.6] months.

There was a significant difference in the socio-emotional subset scores before and one, three, six and twelve months after surgery (F= 22.4, *p* = 0.00). The mean physical score at twelve months [78.1] was slightly lower than the score before surgery [80.3], but no significant difference was found between these scores (W= 0.27, *p* = 0.78). However, the scores at one [63.2], three [68.4] and six [74.3] months were significantly lower than before surgery.

The overall UW-QOL score was analyzed by combining the physical (functional status) and socio-emotional (quality of life) subcategories. There was a significant difference in the total UW-QOL scores before and one, three, six and twelve months after surgery (F= 47.5, *p* = 0.00). The mean UW-QOL score at twelve months [77.9] was lower than the score before surgery [86.1], but it was higher that the scores at one [62.6], three [65.1] and six [71.5] months.

**Figure 1:**
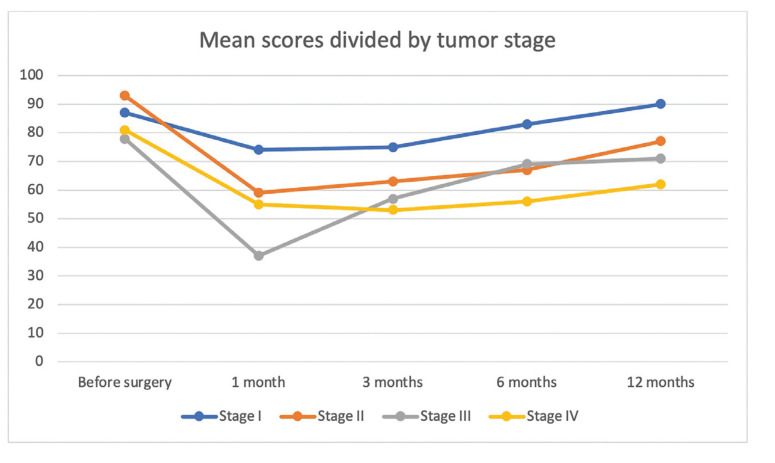
Comparison of mean scores of UWQOL questionnaire stratified by tumor stage.

Fig. 1 summarizes the mean UW-QOL scores divided by tumor stage. Patients with stage I tongue cancer had a mean score of 87.2 before surgery, 74.3 points after one month, 75.6 after three months, 83.4 after six months and 90.7 after twelve months. Patients with stage II tongue cancer had a mean score of 93.2 before surgery, 59.4 points after one month, 63.1 after three months, 67.8 after six months and 77.5 after twelve months. Patients with stage III tongue cancer had a mean score of 78.2 before surgery, 37.3 points after one month, 57.6 after three months, 69.4 after six months and 71.7 after twelve months. Patients with stage IV tongue cancer had a mean score of 81.2 before surgery, 55.3 points after one month, 53.6 after three months, 56.4 after six months and 62.7 after twelve months.

Comparing the mean scores before surgery and after twelve months, only stage I patients had a higher score after twelve months. The percentage of variation in UW-QOL scores one month after surgery was of -15.0% in stage I, -36.0% in stage II, -53.0% in stage III and -32.0 % in stage IV. After twelve months, the percentage of change was of +4.0% in stage I, -17.0% in stage II, -8.0% in stage III and -23.0% in stage IV. Mean scores after twelve months were significantly different between stage I to stage IV patients, with better scores in patients of earlier stages.

**Table 2:**
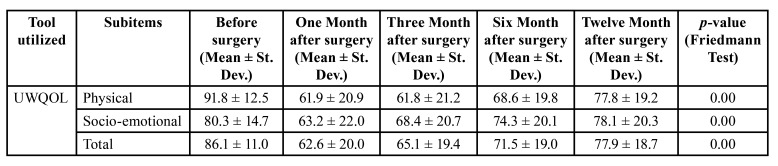
Comparison of UW-QOL scores before and after one, three, six and twelve months after surgical treatment.

**Figure 2:**
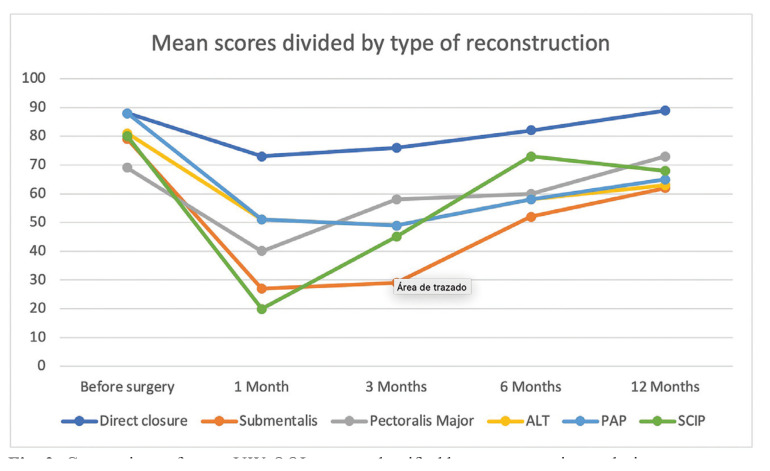
Comparison of mean UW-QOL scores classified by reconstruction technique.

Fig. 2 summarizes the mean UW-QOL scores divided by reconstructive techniques. Direct closure showed the best results throughout all follow-ups, with an average score of 89 at the 12-month follow-up. The rest of the reconstructive methods had poorer scores, especially at the 1- and 3-month follow-up. However, all of them showed a steady improvement at the 6- and 12-month follow-up, with a score range of 62 to 73 at the 12-month follow-up. There was no statistically significant difference between reconstructive methods (other than direct closure).

Table 3 summarizes the coefficients from the stepwise regression model of multiple linear regression analysis. These models predict the factors that influence quality of life and functional status at five different times points: before the surgery, one month after, 3 months after, 6 months after and 12 months after the surgery. The use of UWQOL scores as dependent variables revealed the following predicting factors: age, tobacco use, radiotherapy, chemotherapy, reconstruction method and neck dissection.

Age was a predicting factor in the first month, 6 months and 12 months after surgery, with patients over the age of 69 years showing a significantly worse quality of life. The consumption of tobacco was a predicting factor for patients 1 months after surgery, however it was not significant in the rest of follow-up consultations. Radiotherapy was a negative predicting factor for patients at the 3, 6 and 12 months follow up, with patients that had undergone radiotherapy reporting lesser quality of life parameters. Chemotherapy was a negative predicting factor for patients before surgery and at the 6- and 12-month follow-up. The reconstructive method was a predicting factor for patients at the 1- and 3-month follow-up, however it was not significant for the next follow-ups. Finally, neck dissection was a predicting factor at the 1- and 3-month follow-up.

**Table 3:**
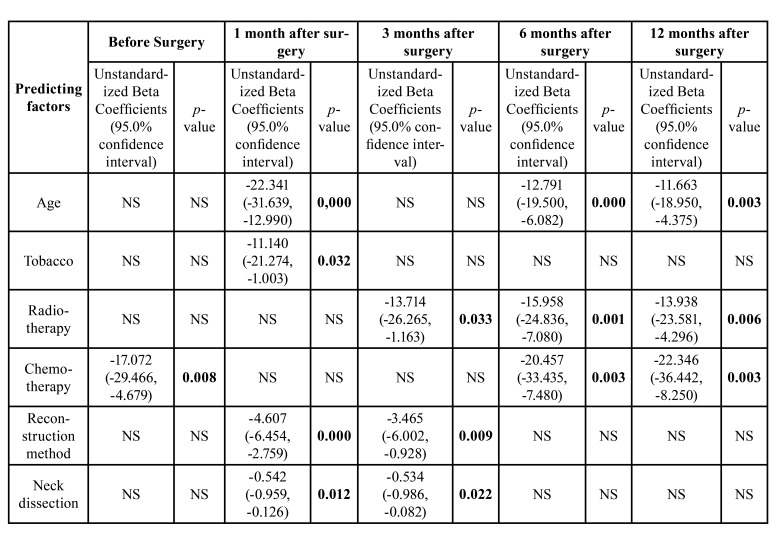
Coefficients from the stepwise regression method of multiple linear regression analysis models for factors predicting the quality of life and functional status.

## Discussion

The goal of our study was to determine the main factors that influence quality of life in patients with tongue cancer that undergo surgical treatment. We found 6 factors that significantly influence quality of life at some point of the treatment; these were: age, tobacco consumption, radiotherapy, chemotherapy, reconstructive method and type of neck dissection. One of the most interesting findings, from a surgical point of view, is that the reconstructive method was a decisive factor in the first 3 months of the postoperative period, but was no longer a significant factor after 6 months. According to our research, this is the first study with a significant follow-up period (12 months) that compares different reconstructive methods in tongue cancer patients. Similar studies (6,9,10) determined that age, tobacco use, and adjuvant therapies were significant factors, which we have also found. However, our study suggests that different types of reconstructive methods were not significant for long term quality of life (11).

Our study population consisted of 36 eligible patients, with a slightly greater ratio of women (55%) compared to men. Our strict inclusion criteria were a limiting factor in being able to collect a larger study group. One of the main factors of rejection was lack of 12-month follow-up. Tobacco and alcohol consumption was present in a significant portion of the study participants. Our study population is similar to those in other comparable studies in age, gender distribution and toxic habits (9-12), however our population did not present any betel nut consumption (which is attribuTable to cultural differences) (6).

Unlike other reports (12-14), our study population included tongue cancer patients of different tumor stages. This permits us to compare quality of life between patients in different stages and relate the effect of different surgical reconstruction methods. Our patient group required radiotherapy postoperatively in 55.6% and chemotherapy in 8% of patients, these Figures are comparable to other similar studies (6,11,12).

Tumor size and size of resection were comparable to other studies (10-15), although most authors don’t mention these Figures specifically. Half of our patients had a small tongue tumor (T1, <20mm) which were treated with partial glossectomy (63,2% with resections under 50 mm). We found that the size of the resection influenced the surgeon’s decision to do a direct closure of the defect or reconstruct with a free flap. An estimation of the glossectomy size limit before the use free flaps became necessary is 45 mm. In our study population all patients that had a direct closure reconstruction had resections under 45 mm. However, the size of the resection was not a determining factor of quality of life. It is our belief that direct closure reconstruction offers a better quality of life outcome over free flap reconstruction, as long as the size of the defect is under 45 mm. Nonetheless, more research is necessary to confirm this hypothesis.

Tongue reconstruction using free or pedicled flaps was necessary in 45.4% of patients, all of which had resections over 45 mm. Free flaps were used as the primary reconstructive method, with the PAP flap being the most common (5). Pedicled flaps were used in case of failure of the primary free flap, with the Pectoralis major flap being the most common. The reconstructive method influenced quality of life in the 1-month and 3-month follow-up consultation, with direct closure patients scoring higher than tongue reconstruction patients. However, this factor was not significant in the 6-month and 12-month follow-up. It must be taken into account that direct closure patients presented smaller tongue defects, so the direct closure and tongue reconstruction sub-categories are not comparable. Therefore, we can assess that the type of reconstructive method does not influence quality of life in the long term. Also, the free flap type (PAP, ALT, SCIP) did not present significantly different scores throughout the follow-up period.

The UWQOL scores were divided into physical and socio-emotional subsets (7). The physical subset demonstrated a significant decrease of scores in the 1-month and 3-month consultations. However, they increased in the 6-month follow-up and again in the 12-month consultation. Comparing the score of the pre-surgical consult and 12-month follow-up we find that the scores are significantly different, so the quality of life was not fully regained. The socio-emotional subset demonstrated a significant decrease of scores in the first 3 months after surgery, with a steady improvement over the next months. Comparing the score of the pre-surgical consult and 12-month follow-up we find that the scores are not significantly different, which suggests almost complete recovery of socio-emotional factors. Taking into account the overall UWQOL scores we find that there is a worsening of the functional status during the first three months, followed by a steady improvement after twelve months after surgery. However, overall quality of life was not regained after 12 months of surgery.

When we stratify our study population by tumor stage, we find that the UWQOL scores and consistently lower as the tumor stage increases. In the pre-surgical consult, the scores ranged from 87.2 to 78.2 (mean score 86.1± 11.0). The scores fell in the 1-month follow-up to a range of 74.3 to 37.3 (mean score 62.6 ± 20.0). From this point forward, almost all scores improved in the next months. The only exception was stage IV patients that presented a worse score in the 3-month follow-up, probably due to adjuvant radiotherapy and chemotherapy. At the 12-month follow-up all patient subsets showed improved scores. Only patients with stage I tumors presented a better mean score at 12-month compared to pre-surgical mean scores.

When we divide our study population by reconstructive technique, we find that patients treated with direct closure of the resection showed consistently better scores throughout all follow-up consultation. However, this subset of patients had significantly smaller glossectomy defects and are therefore not comparable to the rest of the patient population. When evaluating the rest of patients, we see that the scores have a more homogeneous evolution. The scores fell to their lowest levels at the 1-month a 3-month follow-up, with no significant differences between these two. However, the scores improve gradually at the 6-month and 12-month consults. The scores at 12-month showed no significant differences between different reconstructive techniques. This suggest that there is no difference between free and pedicled flaps, as well as different types of free flaps. However, we cannot compare these scores with patients who had not undergone reconstruction in advanced stages.

Multiple linear regression analysis determined six significant predicting factors to UWQOL scores at some point of the study. These were age, tobacco consumption, radiotherapy, chemotherapy, reconstructive method and neck dissection. Age was a factor that showed significant influence on UWQOL scores in the 1-, 6- and 12-month follow-up, with scores worsening with increased age (patients over 69 years of age had significantly lower scores that younger patients). Tobacco consumption was a predicting factor in the 1-month follow-up, perhaps due to conditions associated with smoking (16,17) (emphysema, COPD, etc.) that worsened quality of life when patients were hospitalized and/or required tracheostomy.

Radiotherapy was a determining factor beginning at the 3-month follow-up and lasting for the rest of the study. This could be because the radiotherapy protocol started on average 6 weeks after surgery, and this decreased quality of life due to the side effects associated with this treatment (18). Chemotherapy was a determining factor at the pre-surgical consultation and then again starting at the 6-month follow-up onwards. This could be because patients that required this treatment presented advanced-staged disease before surgery (19). Afterwards, the side effects associated with chemotherapy justify the worsening scores after 6 months, in a similar way to radiotherapy.

As previously discussed, reconstructive techniques were a determining factor in the 1- and 3-month follow-up but not in further follow-ups. This could be because patients that require tongue reconstruction with free or pedicled flaps showed significantly lower scores in the first months compared to direct closure. This could be due to the larger size of resection and consequent worse function of the tongue (20,21). However, after 6 months patients that had undergone tongue reconstruction showed improved function and therefore the differences between direct closure and patients that required reconstructive flaps were not meaningful.

The last predicting factor of UWQOL scores was neck dissection. This was significant at the 1- and 3-month follow-up but did not remain a substantial factor in further consultations. Patients that had sentinel node biopsy had better outcomes due to the fact that the surgery is far less invasive than standard neck dissection, with most patients that underwent this surgery being able to go home after only 2 days (78,0%). Cervical sentinel node biopsy has been proven to be equally effective in finding affected lymph nodes in oral cancer (22). However, our study is the first that describes improved quality of life in patients that underwent cervical sentinel node biopsy compared to neck dissection.

There are several limiting factors in this study. The number of study participants was limited due to the strict inclusion and exclusion criteria and the fact that our target population is limited to the provinces of Cordoba and Jaen. A continuation of the study in a multi-center format could increase the patient population. Another limiting factor is that we are not able to compare quality of life between patients that had large glossectomy defects and tongue reconstructions versus those who did not have reconstruction. However, this is a difficult comparison to make in our patient population for ethical reasons. We propose further research topics that derive from this study. The quality of life of tongue cancer patients requires a focused approach on the surgeons’ part, not only in the operating theater but also in the postoperative period since rehabilitation programs (breathing, swallowing and speech) are a key part in regaining as much function as possible.

## Conclusions

Quality of life outcomes is a parameter that is becoming more relevant in head and neck cancer. When it comes to tongue cancer, new efforts are being done to reconstruct the tongue whenever possible. We have found that flap reconstruction becomes increasingly necessary when a glossectomy resection is over 45 mm, in order to maintain tongue function. We established that the reconstructive flap type does not influence quality of life in the long term. Also, we have found that cervical sentinel node biopsy provides better quality of life over neck dissection in the first 3 months after surgery.
